# Fundus Autofluorescence and Clinical Applications

**DOI:** 10.18502/jovr.v16i3.9439

**Published:** 2021-07-29

**Authors:** Cameron Pole, Hossein Ameri

**Affiliations:** ^1^Retina Division, USC Roski Eye Institute, Keck School of Medicine, University of South California, Los Angeles, CA, USA

**Keywords:** Fundus Autofluorescence, Fundus Camera, Near-infrared Autofluorescence, Retinitis Pigmentosa, Scanning Laser Ophthalmoscope, Short-wave Autofluorescence

## Abstract

Fundus autofluorescence (FAF) has allowed *in vivo* mapping of retinal metabolic derangements and structural changes not possible with conventional color imaging. Incident light is absorbed by molecules in the fundus, which are excited and in turn emit photons of specific wavelengths that are captured and processed by a sensor to create a metabolic map of the fundus. Studies on the growing number of FAF platforms has shown each may be suited to certain clinical scenarios. Scanning laser ophthalmoscopes, fundus cameras, and modifications of these each have benefits and drawbacks that must be considered before and after imaging to properly interpret the images. Emerging clinical evidence has demonstrated the usefulness of FAF in diagnosis and management of an increasing number of chorioretinal conditions, such as age-related macular degeneration, central serous chorioretinopathy, retinal drug toxicities, and inherited retinal degenerations such as retinitis pigmentosa and Stargardt disease. This article reviews commercial imaging platforms, imaging techniques, and clinical applications of FAF.

##  INTRODUCTION

Fundus autofluorescence (FAF) imaging is based on detecting physiologically and pathologically occurring fluorophores primarily in the photoreceptors and retinal pigment epithelium (RPE) to map the metabolic profile of the fundus. Originally described for *in vivo* retinal imaging by Delori et al,^[1]^ autofluorescence (AF) relies on the physical concept of fluorescence in which a molecule absorbs a photon of specific excitation wavelength, undergoes molecular energy transformations, and emits a lower energy photon of specific emission wavelength. The same imaging concepts from fluorescein angiography (FA) and indocyanine green angiography (ICGA) apply to FAF imaging, except the substances imaged are fluorophores already present in retinal structures instead of a systemically administered dye. The AF signals generally must be amplified due to much lower fluorescence than the dyes and may require additional wavelength filters, light path modifications, or detectors. This review aims to provide a clinically relevant overview of ophthalmic fluorophores, commercial imaging platforms, and updated clinical applications for selected conditions.

##  METHODS

Several literature searches were conducted prior to and during the drafting of this review. Both PubMed and Google Scholar were queried using the following search terms: fundus autofluorescence, short wave autofluorescence, near infrared autofluorescence, lipofuscin, scanning laser ophthalmoscope, fundus camera, wide-field autofluorescence, quantitative fundus autofluorescence, fluorescent lifetime imaging ophthalmoscopy. These were used alone, as well as in combination with the following terms: age-related macular degeneration, geographic atrophy, exudative macular degeneration, retinal pigment epithelial tears, subretinal drusenoid deposits, reticular pseudodrusen, central serous chorioretinopathy, diabetic retinopathy, diabetic macular edema, Stargardt disease, macular dystrophy, fundus flavimaculatus, pattern dystrophy, multifocal pattern dystrophy, adult-onset foveomacular vitelliform dystrophy, reticular pattern dystrophy, fundus pulverulentus, butterfly dystrophy, Best macular dystrophy, autosomal recessive bestrinopathy, *ABCA4* dystrophy, retinitis pigmentosa, rod-cone dystrophy, Bietti crystalline dystrophy, CRB1 autofluorescence, pigmented paravenous retinochoroidal atrophy, macular telangiectasia, pseudoxanthoma elasticum, hydroxychloroquine, deferoxamine, pentosan polysulfate, retinal detachment, and retinoschisis. Reviews were referenced if original claims were made, but references to original data in the review were referenced themselves. Data for imaging systems were obtained from either official company websites or user manuals or information from company representatives. The World Health Organization website was used to reference statistics and to confirm published claims.

##  RESULTS

### Fundamentals of Autofluorescence

#### Molecular origin

Fluorescence is an electronic process occurring at the atomic, molecular, or nanostructural level.^[2]^ First, an incident photon is absorbed by electrons in the fluorophore, which is elevated to an excited state. Second, the fluorophore rapidly dissipates some of the absorbed energy through transfer primarily either to its surroundings via kinetic energy or to other vibrational states within the same molecule. The electrons in the molecule may relax to several different vibrational states during this period. Last, when this vibrational energy level overlaps with specific molecular energy levels, the excited electrons may transition to a different vibrational level in a lower electronic level or to its ground state. During this transfer, a photon of energy particular to the electronic structure of the molecule may be emitted. Due to the multiple transfers of energy and natural entropy, the emitted photon is always of a lower energy than the absorbed photon. The excitation wavelength of the incident photon is in turn always shorter than that of the emitted photon.

When the fluorophore is polyatomic, the electronic transitions encompass excitation and emission spectra of a broad band of wavelengths. However, each molecular spectrum has a wavelength maximum at which the fluorophore results in greatest intensity of fluorescence. Similarly, the emission spectra has a wavelength of maximum intensity emission. The excitation and emission spectra wavelengths may overlap, which leads to a resultant decrease in contrast with imaging. This can be ameliorated with selection of appropriate excitation and emission filters to select desired wavelengths and remove overlapping wavelengths of lower intensity.

**Figure 1:**
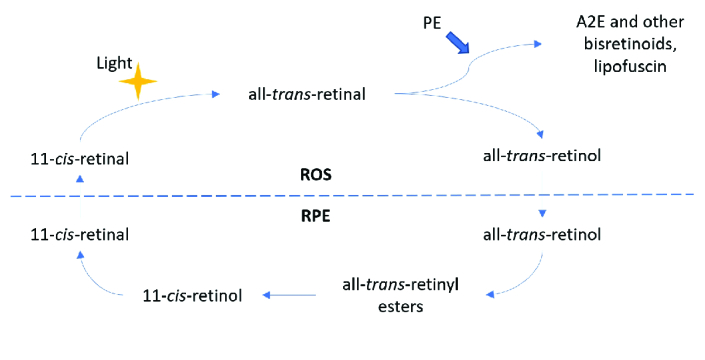
Simplified diagram of the visual cycle and formation of lipofuscin. PE, phosphatidylethanolamine; A2E, N-retinyl-N-retinylidene ethanolamine. The dotted line divides processes occurring in the rod outer segments (ROS) and the retina pigment epithelium (RPE).

Lipofuscin (LF) is a cellular metabolic by-product resulting from incomplete lysosomal degradation of shed photoreceptor outer segments by the RPE and represent the primary substance imaged with short-wavelength autofluorescence (SW-AF).^[3]^ At the start of phototransduction, 11-*cis-*retinal chromophore of rhodopsin absorbs a photon of light and isomerizes to all-*trans-*retinaldehyde [Figure 1]. This form may be released from the activated rhodopsin and react with phosphatidylethanolamine (PE), also in the disc membrane, to form *N-*retinylidene-PE (NRPE). Another molecule of all-*trans-*retinal often escapes conversion to the alcohol form and combines with a molecule of NRPE.^[4]^ This intermediate is then taken up by the RPE and converted to N-retinylidene-N-retinylethanolamine (A2E), a stable pyridinium bisretinoid and the most well-known LF component.^[5]^ When subjected to blue light, A2E is photo-oxidized and generates reactive oxygen intermediaries that can destabilize cellular membranes, interfere with cholesterol metabolism, and trigger apoptosis.^[6,7,8]^

**Figure 2:**
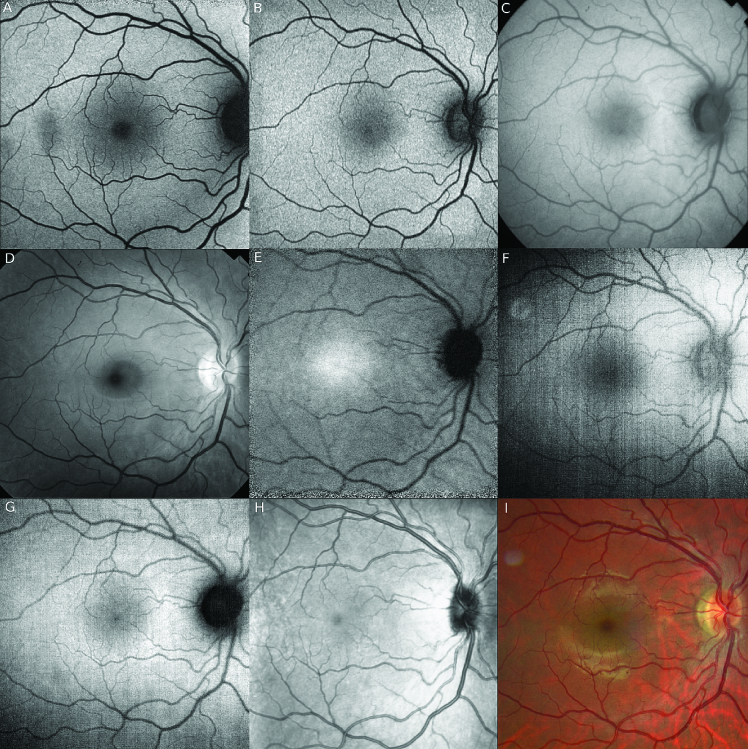
Different autofluorescence techniques imaging a normal right fundus. (A) Blue short-wave autofluorescence (SW-AF), Spectralis (488 nm excitation), scanning laser ophthalmoscope (SLO). The temporal macular hypo-autofluorescence is artifact from a vitreous floater. (B) Green SW-AF, Optos (532 nm excitation), SLO. Note the foveal center is less hypo-autofluorescent (hypo-AF) than in (A). (C) SW-AF, Topcon (535–580 nm excitation), Fundus camera. (D) Red-free image, Topcon. (E) Near-infrared autofluorescence (NIR-AF), Spectralis (787 nm excitation), SLO. Note the fovea is hyper-autofluorescent (hyper-AF) compared with other AF modalities. (F) Blue SW-AF, Clarus 700, Zeiss (435-500 nm excitation). (G) Green SW-AF, Clarus 700, Zeiss (500–585 nm excitation). (H) Near-infrared reflectance image (NIR-R), Spectralis (787 nm) SLO. Note: Neither (D) nor (H) are true AF modalities.

**Figure 3:**
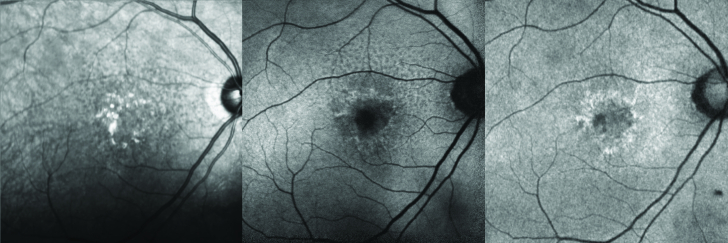
Comparison of near-infrared reflectance (NIR-R), blue-light, and green-light short-wave fundus autofluorescence (SW-AF) images of the right eye in a patient with dry age-related macular degeneration. Left: NIR-R (Spectralis) image. Middle: Blue light (Spectralis) SW-AF image. Note the hypo-autofluorescence of the optic disc and the foveal center. Right: Green light (Optos) SW-AF image. Note the less hypoautofluorescent appearance of the optic disc and foveal center. The subretinal drusenoid deposits are readily visible with the NIR-R and blue SW-AF while poorly visible with green light SW-AF.

**Figure 4:**
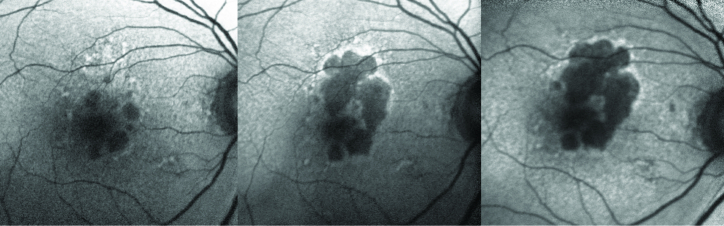
Progression of dry age-related macular degeneration on blue light (Spectralis) fundus autofluorescence (FAF). Left: Right eye of a patient with geographic atrophy (GA). Note the hypoautofluorescent (hypo-AF) patches with superior hyperautofluorescent (hyper-AF) lesions. Middle panel: Progression of the GA superiorly into the area of previous hyper-AF. Note the hyper-AF border of the GA indicating areas of disease activity. Right: Further progression of GA, primarily at the edges with prior hyper-AF.

**Figure 5:**
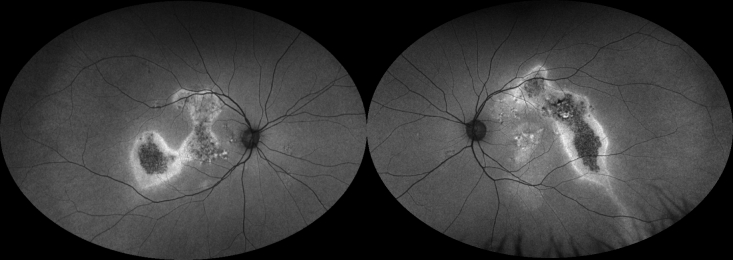
Green light ultrawide field fundus autofluorescence (UWF-FAF) (Optos) of the fundi of a patient with chronic central serous chorioretinopathy (CSCR). Left: Right eye demonstrating patches of mottled hyperautofluorescence (hyper-AF) and hypoautofluorescence (hypo-AF) indicating variable retinal pigment epithelium (RPE) and outer retinal atrophy. The surrounding hyper-AF indicates outer retinal thinning with intact RPE. Right: Left eye demonstrating similar findings to the right eye, with more prominent gravitational pattern, which is diagnostic.

**Figure 6:**
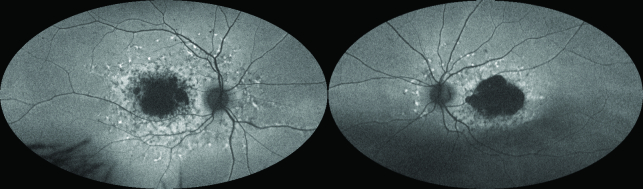
Green light ultrawide field fundus autofluorescence (UWF-FAF) (Optos) of a patient with Stargardt disease. Note the dense foveal hypoautofluorescence (hypo-AF), retinal pigment epithelial (RPE) atrophy. Perimacular hyperautofluorescent (hyper-AF) flecks comprised of lipofuscin buildup extend toward the mid-periphery. Hypo-AF flecks likely indicate prior lipofuscin deposits that resulted in RPE atrophy. Peripapillary sparing of these deposits is characteristic of Stargardt disease.

**Figure 7:**
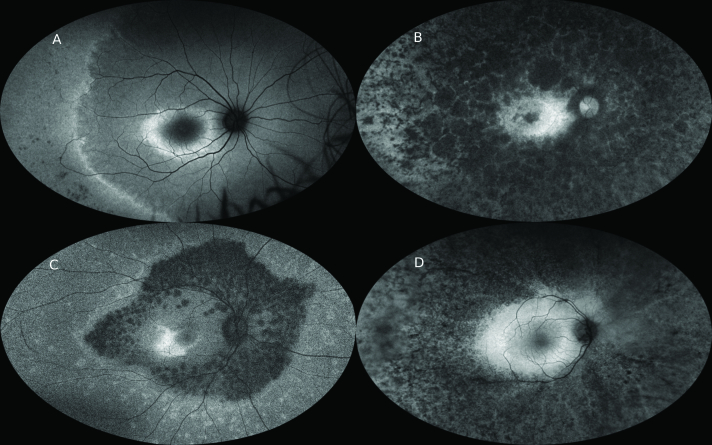
Green light ultrawide field fundus autofluorescence (UWF-FAF) (Optos) imaging of different patients with retinitis pigmentosa (RP). (A) Note the macular hyperautofluorescent (hyper-AF) ring and a second hyper-AF ring in the periphery. Pigment clumping can be seen in the inferotemporal periphery. (B) Note the dense peripheral nummular hypoautofluorescence (hypo-AF) indicating retinal pigment epithelial (RPE) atrophy. There is no obvious hyper-AF macular ring. (C) Note the abrupt demarcation from dense hypo-AF mottling to more uniform mild hypo-AF peripherally. There is an asymmetric hyper-AF macular ring. (D) Note dense peripheral speckled hypo-AF with preserved macular AF. There is no distinct macular hyper-AF ring. Note that in (A) and (C), despite widespread peripheral photoreceptor loss and dense visual field constriction clinically, in there is preservation of some FAF signal peripherally and therefore incomplete RPE loss.

**Figure 8:**
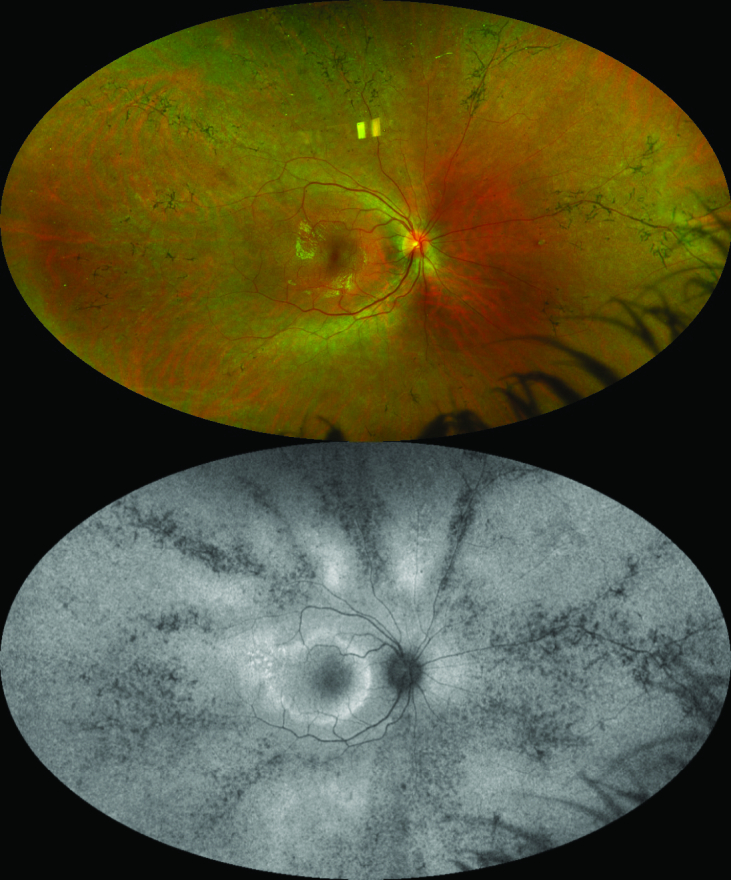
Fundus imaging of a patient with pigmented perivenous retinochoroidal atrophy (PPRCA). Top: Pseudocolor ultrawide field (UWF) imaging of the right eye (Optos). Note the bone spicule pigment clumping and chorioretinal atrophy around the retinal veins. Bottom: Green light UWF fundus autofluorescence imaging of same eye (Optos). Note more obvious hypoautofluorescent (hypo-AF) changes in the periphery than can be seen on the color imaging. A hyperautofluorescent (hyper-AF) macular ring can be noted.

Combinations of at least 20 compounds formed during incomplete visual pathway biosynthesis or subsequent photooxidation may form LF granules.^[9,10]^ These compounds may then cross-link with themselves and other intracellular molecules to form complex end-products.^[11,12]^ Photooxidative processes affecting cellular proteins and membrane lipids likely contribute to the LF milieu. Proteomic studies of purified LF granules identified A2E adducts in addition to an abundance of photo-oxidated proteins and lipids, with only 2% by weight of the LF being amino acids.^[13]^

Moreover, although the fluorescent products of lipid oxidation maximally emit light in the blue–green spectrum,^[14,15]^ RPE LF extracts have consistently emitted light in the yellow–orange spectrum.^[10,16]^ Debate continues on the origin of these emissions and whether measuring them clinically is useful.

#### Wavelengths

The various bisretinoids making up LF are the primary source of the retina AF signal noted with SW-AF.^[1,17]^ They have a peak excitation in the blue range ( ∼ 470 nm) resulting in emission with a peak in the yellow–orange range (600–610).^[17]^ The absorbance maximum that have been identified include 430 nm (all-*trans-*retinal dimer), 439 nm (A2E), 449 nm (A2PE), 426 nm (isoA2E), 490 nm (A2-DHP-PE), and 510 nm (all-*trans-*retinal dimer conjugates).^[18,19,20]^ Therefore, although individual bisretinoids may fluoresce with blue 488 nm FAF excitation, their different absorbance spectra lead to different emission intensities. Moreover, all-*trans-*retinal and its ester, physiologic intermediaries during phototransduction, do not contribute significantly to the FAF pattern at 488 nm excitation.^[17]^

First detected during FA as preinjection fluorescence, normal fundus SW-AF signal demonstrates a dark area centered on the fovea, dark vessels strikingly contrasted against a bright retinal background, and a dark optic disc. The foveal pigments, including lutein and zeaxanthin, reach a maximum concentration at the fovea and decreases exponentially with eccentricity. These pigments are primarily concentrated in the photoreceptor layer axons but can be found in the inner plexiform layer.^[21]^

Near-infrared FAF (NIR-FAF) was first detected while evaluating pre-ICG injection fluorescence with standard ICG angiography filters.^[22]^ Normal fundi increased NIR-FAF signal in the fovea corresponding to a higher melanin concentration and the luteal pigment [Figure 2]. The maximum NIR-FAF signal in the fovea is very slightly nasal to the maximum SW-FAF signal, and it is always of lower contrast and exposure to the SW-AF signal, even with significantly higher radiant power and detection sensitivity,^[23]^ and may require dilated pupils for sufficient image quality. The optic disc and retinal vessels have dark signal similar to SW-AF images but with less contrast compared to the surrounding retina. Melanin in the RPE and choroidal layers contribute to NIR-FAF that can be noted with RPE hyperpigmentation, choroidal nevi,^[24]^ and the outline of choroidal vessels,^[23]^ which appear dark. Subacute blood, orange–yellow deposits or chronic subretinal fluid over choroidal melanomas or nevi, and some choroidal neovascular membranes (CNVM) can be bright on NIR-FAF.^[22]^

Near-infrared reflectance (NIR-R) utilizes an infrared light source ( ∼ 820 nm) and detector to image the retina and will not be extensively discussed in this review.

In this review, AF will refer to SW-AF unless specifically noted otherwise for NIR-FAF.

### The Autofluorescence Signal

AF was first reported in the 1970s, when autofluorescent signal of optic nerve head drusen was noted prior to the injection of fluorescein dye.^[25]^ Additional FAF was noted soon thereafter with retinal astrocytic hamartoma and Best disease (BD).^[26]^ Histopathology confirmed these findings, with diseased RPE noted to be more highly fluorescent than normal RPE on whole flat mounts.^[27]^ However, accumulation of A2E-related compounds and other fluorophores can occur posterior to and in many structures anterior to the RPE.

Areas of increased SW-AF, or hyper-AF, result from buildup of AF compounds, an unmasking of these compounds from depleted photoreceptor or luteal pigments, and/or presence of structures that also autofluoresce at the excitation wavelength. Areas of decreased SW-AF, or hypoautofluorescence (hypo-AF), result from loss of AF compounds or RPE, or blockage from overlying substances or structures. Increased SW-AF can be noted with porphyrins from subacute to chronic retinal hemorrhage,^[28]^ disruption of luteal pigment causing unmasking of foveal AF,^[29]^ bare sclera, calcification, optic disc drusen, or any condition that increases LF accumulation and/or migration, such as BD, STGD, and the pattern dystrophies.^[29,30]^

Molecules in the pathway of incident light and/or the emitted photons can interfere with the detected signal. Rhodopsin chromophores in the photoreceptor outer segments filter the excitation and emission light, but may also contribute to the FAF signal in certain retinal pathologies.^[31]^ Increased FAF signal from the RPE can be achieved by “bleaching” of the photoreceptor pigments with high intensity light and photoisomerization.^[32]^ Bleached rhodopsin can lead to up to 30% increased FAF signal compared with its dark-adapted state,^[33]^ indicating the importance of the photoreceptor layer in interpretation of FAF.

The crystalline lens contributes to absorption of excitation and emitted light.^[34]^ Importantly, it filters lower wavelength light (292–400 nm) to prevent retinal damage from ultraviolet light. However, increased post-translational modifications to lens proteins during aging shifts the absorption maxima more into the visible range.^[35]^ This yellowing of the lens results from covalent modifications to the protein chain, oxidation of aromatic amino acids, and development of advanced glycation end products.^[36]^ In addition, intrinsic lens fluorescence in the UV-A and UV-B ranges increases with age, although likely contributes less to artefacts in FAF.^[35]^

The human cornea has an excitation peak at 365–480 nm and an emission peak maximum at 620 nm, although it can have increased AF in the visible range in diabetic patients.^[8]^

### Quantitative Autofluorescence (qAF)

Although abnormal AF signal may be readily apparent, subtle differences from normal or progressive changes on subsequent visits may be qualitatively undetectable. A need to standardize and quantify FAF signal for reproducible and comparable measurements led to the development of qAF.

Gray pixel values for FAF on imaging systems are normally assigned a value from 0 to 255 but can vary depending on system calibration. Delori et al normalized a FAF system to a standard reference and compensated for factors such as laser power, refractive error, and media opacity variations, in order to calculate the qAF value from the gray level measurements.^[37]^ Physiologically higher qAF levels have been demonstrated with increasing age, increasing eccentricity up to 15° from the fovea, the superotemporal fovea, and females.^[38]^ Levels are also higher in Whites compared with Blacks and Asians. To obtain accurate measurements, it is critical to have high-quality images with a skilled ophthalmic photographer, dim and usual lighting, ideal focus, and consideration of significant media opacities that may confound interpretation.^[9,38]^ Good inter-operator agreeability and between-session repeatability have been shown with trained technicians and standardized imaging procedures.^[38,39]^

### Fluorescence Lifetime Imaging Ophthalmoscopy (FLIO)

As opposed to qAF which measures the standardized intensity of fluorescence, FLIO measures the time delay from excitation to emission and the decay curve of emission intensity, which are not necessarily related to fluorescent intensity.^[9]^ Every fluorophore harbors a unique excitation and emission spectra as well as a fluorescence lifetime.^[40]^ Quantifying the time from excitation to emission can be measured directly or by modulating flicker light and measuring the phase delay of emission. Problems with the complexity of measuring the decay at each pixel in an image with computational exponentials can be overcome by transforming the histogram of time delays at each pixel into a phasor.^[41]^ The details of this imaging are beyond the scope of this review, but this approach can allow identification of molecular species at each pixel and their proportions. In healthy eyes, increased AF lifetimes have been noted with age^[42]^ and increased distance from the fovea,^[43]^ likely from macular pigment distributions. In general, a significant increase in lifetime is seen in diseased retina and RPE, often due to the buildup of LF with its relatively long lifetime.^[40]^ Disease-specific changes have been noted with AMD,^[44]^ macular telangectasia type 2 (MacTel),^[45]^ Stargardt disease (STGD),^[46]^ hydroxychloroquine retinopathy,^[47]^ and choroideremia.^[48]^ While clinical utility is currently limited, results of these studies are encouraging for monitoring disease course for observation and therapeutic trials.

### Imaging Systems 

The design of the first scanning laser ophthalmoscope (SLO) was first presented in 1980^[49]^ but has evolved significantly over the years.^[50]^ All SLO systems utilize a laser source to generate a “flying” laser spot that passes through a series of tunable mirrors and lenses to scan horizontally and vertically in a raster pattern over the retina. At each point, the backscattered light from the retina is directed into a detection arm for analysis by software and image creation. Advantages of SLO include lower required light intensity, high frame rate, increased sensitivity, and smaller aperture, that is, smaller pupil, requirements. Disadvantages include the lack of true color imaging, artefacts from eye movements, and spatial distortions.

However, some modern systems implement confocal components, which allow imaging of specific planes in the pathway of the laser. By modifying the position of the mirrors and directing the light through a pinhole, the photodiode detector can amplify reflected light from the desired plane of focus while blocking out-of-focus light.^[51]^ Suppression of out-of-focus light increases at further distances from the focal plane; thus, signal from vitreous opacities, lens aberrations, and the cornea are minimized. This provides improved 2D resolution and contrast as well as 3D imaging capabilities.

Fundus cameras rely on a high-energy white flash that passes through a wide-band excitation filter and series of mirrors and apertures to illuminate the retina. Issues with these systems include low FAF signal, AF of light pathway media, and inherent non-confocality of the system.

Inherent limitations in FAF imaging include low AF signal when compared with FA or color fundus imaging, high image noise, low contrast, and AF artifacts from more anterior structures. Modern systems utilize strategies to increase the true posterior FAF while reducing anterior segment interference.

Modern confocal SLO (cSLO) systems include the Heidelberg Retinal Angiograph (HRA) on SpectralisⓇ platforms (Heidelberg Engineering GmbH, Heidelberg, Germany), the Nidek F-10 and Mirante systems (Nidek, Gamagori, Aichi, Japan), and the EIDON device (Eidon, Centervue, Padova, Italy) [Table 1]. The Heidelberg SpectralisⓇ HRA2 SW-AF utilizes blue light, with a solid state laser at 488 nm for excitation and a > 500 nm filter for emission, and 20–55 degree images.^[52]^ Imaging with NIR-AF is also provided with a 787-nm excitation and > 800 emission filter. As the most widely used commercial device, the HRA spectral domain optical coherence tomograph (SD-OCT) can be combined with the cSLO for simultaneous FAF and OCT recordings, automatic real-time imaging for guided acquisition registration of the fundus by the cSLO, eye-tracking for movement correction, and image tracking for monitoring progression on follow-up visits.^[53]^ The Nidek F-10 and Mirante systems use 490-nm blue light with an emission filter at 510 nm and a 40–60°field of view.^[9,54]^ They also harbor an infrared imaging mode at 700 nm to detect laterally scattered light, which may be more sensitive for drusen detection.^[9]^ The EIDON device by Centervue is a cSLO with FAF capabilities that uses a larger than standard pinhole.^[9]^

The Rodenstock cSLO and Zeiss prototype SM 30 4024 cSLO are no longer commercially available.

The commercially available Zeiss (Carl Zeiss Meditec, Dublin, California, USA), Canon CX-1/CR-2 (Canon, Tokyo, Japan), Topcon TRC-50DX/50IX (Topcon Corporation, Tokyo, Japan) systems and their specifications are listed in Table 1. These traditional-style fundus cameras all incorporate single flashes of light with bandpass excitation filters in the blue–green range and emission filters in the near- infrared range.^[2,9,55,56]^ New red-shifted modifications of the Spaide filters can be swapped in with excitation of 535–585 nm and emission filters of 615–715 nm. These modified filters create lower AF signal from the crystalline lens and cornea. The bandpass excitation filter incorporates wavelengths above that absorption spectra of fluorescein, allowing FAF images to be taken after fluorescein injection.^[57]^

Zeiss produces the Visucam family of retinal cameras, the FF 450 Plus IR device, and the Clarus cameras. For FAF imaging, the Visucam and FF450 use excitation filters of 510–580 nm and emission filters with a bandpass of 650–735 nm. The Clarus 500/700 systems are marketed as a true-color, ultra-wide field system using Broad Line Fundus Imaging (BLFI) technology with illumination in two broad wavelengths, using two excitations at 435–500 (blue) and 500–585 (green) nm and emission filters of 532–650 and 630–750 nm, respectively. The Clarus captures a wide range of SW-AF and can image up to 200° with two montaged images.^[58]^

The Optos (Optos, Dunfermline, United Kingdom) OptomapⓇ ultra-widefield (UWF) imaging platforms are non-mydriatic cSLO with FAF capabilities.^[2,9,59,60]^ A scanning laser is reflected off a concave mirror and rasterized to visualize up to 180–200° of the retina in a single capture. As opposed to the HRA2, a green laser at 532 nm is used for excitation, and an emission filter at 540 nm acquires emitted light in the 540–800 nm range [Figure 3]. The laser and pathway are the same for all current models as well as the earlier 200Tx model. The rapid scanning speed with the ability to capture wide angles in a single image can be advantageous in patients with poor vision or poor fixation. An additional advantage of UWF imaging is improved acquisition ability through small pupils ( ∼ 2 mm) and media opacities. An infinite focal range inherent to the system results in all structures being in focus. Disadvantages include pseudocolor images inherent to other cSLOs, and horizontal stretching of images and peripheral enlargement, leading to unreliable measurements, but recent advances have employed algorithms to compensate for these distortions.^[61]^

**Table 1:** Commercially available fundus autofluorescence devices and specifications


**Device(s)**	**Manufacturer**	**Imaging type**	**Excitation wavelength (nm)**	**Emission wavelength (barrier filter) (nm)**	**Field of view (degrees)**	**Modifications**	**Advantages**
Spectralis (HRA2)	Heidelberg	cSLO	488 (blue, SW-AF), 787 (NIR-AF)	> 500 (SW-AF), > 800 (NIR-AF)	15, 20, 30, 55	Heidelberg UWF lens for 55 degrees	Can be combined with OCT, FA/ICG, NIR-R, multicolor imaging.
F-10, Mirante	Nidek	cSLO	490 (blue SW-AF), 532 (green SW-AF), 790 (Retro-mode)	510 (blue SW-AF), ∼ 630 (green SW-AF)	40, 60, 163	Wide field adapter	Can be combined with OCT, FA/ICG, NIR-R, multicolor imaging. Retro-mode may be useful for imaging drusen.
EIDON	iCare	cSLO	450	510–560, 560–700	60	May detect additional fluorophores, wider range of FAF signal due to full color sensor.
Clarus 500/700	Zeiss	BLFI	435–500 and 500–585	532–650 and 630–750	133, 200 (montaged)	Uses two light sources and filters for broader range of autofluorescence.
Optomap*	Optos	UWF cSLO	532	540–800	200	Can be combined with OCT, FA/ICG, multicolor.
TRC-50DX/50IX	Topcon	Fundus Camera	500–610 (conventional), 535–585 (Spaide filters)	675–715 (conventional), 615–715 (Spaide filters	20, 35, 50	Spaide red-shifted filters	Spaide filters allow improved cataract penetration, less macular pigment blockage, and less light requirements. Can perform FA/ICG prior to FAF imaging.
Visucam 224/524, FF450 Plus IR	Zeiss	Fundus Camera	510–580	650–735	30, 45 (Visucam); 20, 30, 50 (FF450)	FF450 uses black and white Pike145b or 421b filters	Can be combined with FA/ICG, color fundus imaging.
CX-1/CR-2	Canon	Fundus Camera	530–580	> 640	35, 45	Can use cobalt blue setting.
AF, autofluorescence; BLFI, broad line fundus imaging; cSLO, confocal scanning laser ophthalmoscope; FA, fluorescein angiography; ICG, indocyanine green angiography; NIR-AF, near-infrared autofluorescence; NIR-R, near-infrared reflectance; OCT, optical coherence tomography; SW-AF, short-wave autofluorescence; UWF, ultra-wide field. *Optomap AF is available on any of the Optos devices							

The optic nerve SW-AF signal varies depending on both the imaging system and wavelength used. For example, on the Spectralis (488 nm, blue), F-10/Mirante (490, blue; 532, green), and EIDON (450 nm blue), the optic nerve appears very hypo-AF and almost black, but on the Optos (532 nm, green), the nerve appears less hypo-AF and almost isoautofluorescent to the fovea, despite all systems being cSLO systems [Figure 2]. Generally, the optic disc appears hyper-AF on fundus cameras, which are mostly green SW-AF. With the Clarus, the optic disc appears very hypo-AF with green SW-AF (500–585 nm) and isoautofluorescent to retina with blue SW-AF (435–500 nm). The reasons for these discrepancies may be due to the varying focal ranges, excitation and emission barriers, and/or light scattering.

Comparisons between imaging platforms must take into consideration not only cSLO versus fundus camera but also the technical aspects of the machine, wavelength spectra, and the clinical entity being investigated.

### Clinical Applications

#### Age-related macular degeneration (AMD)

Advanced forms of AMD such as geographic atrophy (GA) and neovascular AMD (wAMD) are leading causes of vision impairment worldwide, especially in industrialized countries.^[62]^ As AMD primarily affects the RPE-Bruch's membrane complex, FAF changes, particularly on SW-AF, have been shown to be an extremely useful diagnostic and prognostic tool, even with the advent of high-definition OCT platforms (HD-OCT, which will be used to refer to both spectral domain and swept source OCT in this review).

Early lesions in AMD can be detected more readily with SW-AF than on color imaging as focal hypo-AF and hyper-AF, and a variety of early FAF patterns have been described.^[63]^ Atrophic or degenerating RPE cells with less LF content will be hypo-AF, while hyperplastic or dysfunctional RPE cells with LF accumulation will be hyper-AF. In a study by Bindewald et al,^[63]^ certain AF patterns in early and intermediate AMD were delineated, but drusen were not necessarily correlated with a particular FAF change. Speckled and patchy FAF patterns were most frequent, but FAF changes did not correlate topographically with changes on color fundus imaging. Delori et al^[64]^ noted variable FAF patterns over hard and soft drusen, but with hyper-AF surrounding drusen, suggesting changes in RPE metabolism. Focal vitelliform deposition over drusen may increase FAF signal,^[65]^ but otherwise the intrinsic FAF signal from drusen is variable and cannot be used clinically to differentiate drusen types reliably.^[9]^

Reticular pseudodrusen, or subretinal drusenoid deposits (SDD), represent an independent risk factor for progression to advanced AMD.^[66]^ Although first described on blue light imaging, they are most easily seen on near-infrared reflectance (NIR-R), NIR-AF, and SW-AF but are readily detectable on HD-OCT and red-free imaging.^[67,68]^ They are apparent on IR or AF en face imaging as an interlacing network of small dot-like variations.^[69]^ These correlate to small subretinal hyperreflective deposits on HD-OCT. Thinning of the ONL with disruption of the EZ has been described above the SDD, which may lead to the aforementioned FAF changes.^[70]^ Although not a therapeutic target, SDD should be looked for routinely, as evidence suggests increased risk for progression to GA and association with higher genetic risk scores.^[71,72]^

GA has been shown to be readily and accurately detected and monitored with FAF.^[73]^ The death of RPE and loss of intrinsic fluorophores correlates well with the sharply demarcated borders on FAF^[74]^ and can be correlated with HD-OCT patterns. The pattern of FAF in the junctional zone surrounding the GA has been associated with the rate of GA enlargement, with more diffuse hyper-AF changes generally representing faster rates.^[73]^ The rate of growth on FAF imaging is variable, with large studies ranging from approximately 0.5 to 2.5 mm ${}^{2}$ /year [75, Figure 4]. This rate is positively correlated with bilateral GA and increasing baseline GA area on initial imaging, but negatively with fellow eye early/intermediate AMD status, and without apparent correlation with fellow eye CNV.^[75]^ Multimodal imaging evaluation with VA and tests of visual function noted significant predictors of worse VA with greater areas of confluent hypo-AF and involvement of the fovea with confluent or granular hypo-AF. Larger areas of confluent hypo-AF were also associated with decreased reading speed and contrast sensitivity.^[76]^ Measuring tools on imaging software or vascular landmarks can be used to discern progression of GA.

The utility of NIR-AF is less established than that of SW-AF. Imaging with NIR-AF in early AMD also demonstrates characteristic patterns: normal foveal signal, fovea-sparing hyper/hypo-AF spots, fovea-involving hyper/hypo-AF spots, and a patchy AF pattern.^[77]^ More foveal-involving patterns were correlated with a worse retinal sensitivity on microperimetry (MP). Patterns on SW-AF and NIR-AF may overlap but are not identical,^[78]^ indicating different involvements of LF and melanin in AMD pathophysiology. In the presence of GA, both SW-AF and NIR-AF are comparable in measuring lesion size. However, because SW-AF can be blocked by foveal luteal pigments, SW-AF has been shown to overestimate foveal GA compared with NIR-AF and MP, while NIR-AF overestimates non-foveal GA.^[79]^ The combination of FAF wavelength modalities may provide more accurate measurements, but the clinical utility of the combination remains to be seen.

The Classification of Atrophy Meeting recommended SW-AF adjunctive to HD-OCT in order to monitor the size of GA and detect peri-atrophy AF changes incipient to progression.^[80]^ Evaluation of the fovea in fovea-sparing GA is limited by the absorption of blue light by luteal pigments, which makes the fovea appear dark.^[81]^ Although GA area is quantified reproducibly with either green FAF or blue FAF + NIR, slightly better inter-reader agreement with green FAF was noted in one study.^[82]^ Comparison with HD-OCT is important to assess for exudation, CNV formation, and drusen-associated atrophy, in which outer retinal and RPE atrophy has begun overlying drusen and may be variably autofluorescent.^[83]^

Moreover, wAMD, or macular neovascularization (MNV), has a variable appearance on FAF depending on the integrity of the RPE, the presence of hemorrhage or exudation, and type of MNV. Intraretinal exudates and hemorrhage will block FAF and cause hypo-AF, but with chronicity, hemorrhage may organize and form breakdown products with resultant intense hyper-AF on SW-AF and NIR-AF.^[9,22,84]^ Classic CNV, or type 2 MNV, shows decreased FAF in the area of leakage on FA, while eyes with occult CNV, or type 1 MNV, shows spots of hypo-AF or normal AF.^[85,86]^ As the type 2 MNV lesion is subretinal, it may block the AF signal from the RPE. The type 1 MNV may cause focal RPE atrophy and overlying hypo-AF. No FAF pattern over the MNV has been shown to be predictive of outer retinal integrity.^[85]^ However, the presence of an hyper-AF ring surrounding the MNV prior to injections has been associated with increased baseline SRF, increased number of injections, and greater likelihood of EZ disruption on HD-OCT after SRF resolution.^[87]^ This ring suggests more widespread involvement than apparent on OCT or FA. Eyes with FAF alterations have also been associated with worse initial VA and smaller VA gains after anti-VEGF therapy compared with those without FAF changes.^[88]^ Therefore, while the mainstay of modern wAMD monitoring utilizes HD-OCT, ancillary FAF testing may provide useful prognostics for progression and visual acuity.

Tears of the RPE have been well-documented during both the natural history and treatment of wAMD,^[89,90]^ occurring in approximately 10–12% of patients with wAMD.^[91]^ They traditionally occur in type 1 MNV, with a large, vascularized, and often peaked PED,^[92]^ and contracture of the MNV is thought to produce tensile forces on RPE attachment junction, eventually resulting in tearing of the RPE. As this is a structural complication of the RPE, SW-AF is particularly suited for imaging this feature. The area of Bruch's membrane/bare choroid shows well-demarcated reduced FAF signal, and the adjacent area of scrolled RPE shows increased AF due to folding of the RPE monolayer.^[93]^ Longitudinal FAF imaging demonstrated centripetal reconstitution of the AF signal on the area of bare choroid and suggests recovery by the RPE cells. A grading system of RPE tears has been suggested based on size on FAF and FA (Grade 1 = < 200 µm diameter, Grade 2 = 200 – 1 disc diameter [DD], Grade 3 = > 1 DD, Grade 4 = Grade 3 involving foveal center), and lower grades were associated with better VA and response to continuing anti-VEGF therapy.^[94]^ While studies support continued anti-VEGF therapy in the presence of an RPE tear^[95]^ to improve VA outcomes, progression of the RPE tear has occasionally been noted with continued injections, especially with multilobular RPE tears.^[96]^ Therefore, SW-AF can help diagnose an RPE tear, quantify its extent, provide prognostic information, and guide therapeutic decisions.

As a heterogenous disease, AMD may present with overlapping features of other macular pathologies. Multimodal imaging including FAF can narrow diagnoses. The pisciform flecks of STGD may appear like drusen on color imaging but demonstrate intense hyper-AF with SW-AF, as they are composed of LF deposits. BD and adult-onset vitelliform macular dystrophy also present with various deep yellowish deposits but will be hyper-AF with FAF. Macular dystrophies presenting with drusen-like deposits and RPE changes, such as malattia leventinese/Doyne honeycomb dystrophy, Sorsby fundus dystrophy, central areolar choroidal dystrophy, North Carolina macular dystrophy, cone dystrophies, pseudoxanthoma elasticum, late-onset retinal degeneration, and mitochondrial disorders, can be confused with color imaging.^[97]^ A thorough history-taking, including age of onset and family history, along with multimodal imaging with FAF, and genetic testing, can help distinguish these often phenotypically similar but genetically distinct diseases and guide management.

### Diabetic Retinopathy (DR)

DR affects approximately 35% of the more than 422 million adults suffering from diabetes worldwide.^[98,99]^ Currently, the most commonly used screening and diagnostic modalities for DR include color fundus imaging and FA, both standard and ultrawide field. These provide the most useful detail of DR severity, retinal ischemia, and vascular leakage.^[100]^ However, studies have noted other fluorophores that are involved in cellular metabolism, such as nicotinamide adenine dinucleotide (NADH), flavin adenine dinucleotide (FAD), and advanced glycations end-products (AGE),^[101]^ may affect the FAF signal. Impaired mitochondrial function before pericyte cell death from chronic hyperglycemia alters the cellular balance of these molecules.^[102]^ One study found increased quantitative FAF of flavoproteins in NPDR eyes, suggesting modified retinal metabolomics.^[102]^ Other studies have found more notable DR changes on FAF than color imaging,^[103]^ as well as greater FAF pixel intensity and variation in diabetic patients without DR compared with controls.^[104]^ Additionally, studies have found abnormal FLIO parameters in eyes with NPDR^[105]^ as well as without detectable DR.^[106]^ Both blue and green light sources were used in these studies, so any increased sensitivity of blue imaging must be balanced against interruption from the crystalline lens in phakic patients. Lens and vitreous opacities are common in eyes with DR, and cSLO, particularly UWF, can improve signal penetration and decrease imaging time. The prognostic and therapeutic implications of these findings are still not fully explored, however, and the role of FAF in DR remains investigative.

### Diabetic Macular Edema (DME)

As DR and DME primarily affect inner retinal layers, FAF is less useful than other modalities such as FA or OCT for clinical evaluation. However, FAF can provide useful adjunctive information. Blue macular FAF can increase in the presence of intraretinal fluid, while microaneurysms or blood are more easily visualized with FAF. Foveolar hyper-AF correlated with CME on OCT, and foveal lobules of hyper-AF corresponded to pooling of fluorescein dye on FA.^[107,108,109]^ Increased FAF signal has been significantly correlated with ellipsoid zone (EZ) loss and showed an association with poorer visual acuity, independent on the severity of DME.^[107]^ Another investigation noted increased FAF and diminished MP sensitivity in areas of DME, as well as significant association between abnormal FAF and OCT loci.^[110]^ On the other hand, green FAF has not shown use in identifying DME. A comparative series using UWF green FAF did not show correlation with central retinal thickness (CRT) or the pattern of DME distribution, although macular hemorrhage and microaneurysms could still be visualized.^[60]^

Bessho et al studied FAF with BRVO and DME-related CME and noted abnormal FAF signal with 488 nm excitation but not 580 nm excitation.^[108]^ As Xu et al^[111]^ previously demonstrated that perivascular and subretinal microglia are the primary sources of retinal LF, it was hypothesized that the displacement of macular pigments, which have absorption peaks at 460 nm, by cystoid cavities results in the abnormal signal.

Yoshitake et al studied DME with cSLO NIR-FAF and found a correlation with a mosaic fluorescent pattern and cystoid spaces on SD-OCT, poorer VA, presence of hyperreflective foci, and disrupted ELM.^[112]^ Furthermore, a cystoid-appearing NIR-FAF pattern was related to poorer VA and increased central sub-field thickness (CST) but not to ELM status or presence of hyperreflective foci, compared with eyes without cystoid patterns.

There is limited role for routine FAF for diagnosis and management of DME given the advent of HD-OCT. However, evidence suggests correlations between FAF findings and cystoid changes, visual acuity, and tests of retinal sensitivity, and routine integration into multimodal DME evaluation may allow longitudinal monitoring of outer retinal and RPE integrity.

### Central Serous Chorioretinopathy (CSCR)

CSCR causes idiopathic serous detachments of the retina and RPE, and associations between corticosteroid use,^[113]^ obstructive sleep apnea,^[114]^ type A personalities,^[115]^ and sleep disturbances^[116]^ have been suggested. Both acute and chronic forms of CSCR have been described, with chronic arbitrarily being defined as longer than three to six months. Acute CSCR with serous retinal detachments and focal leakage on FA usually display normal AF signal, while those with more chronic fluid demonstrate moderately increased AF in the area of detachment.^[117]^ In those with years of symptoms, AF over the affected areas can be irregular with both hyper-AF and hypo-AF areas due to variable degeneration of photoreceptor outer segments and RPE. More homogenous hypo-AF is associated with complete RPE atrophy, while hyper-AF corresponds to retinal thinning and outer retinal atrophy with an intact RPE, and is often seen at the border of the hypo-AF patches but can be isolated [Figure 5].^[118,119]^ Granular AF represents either variable RPE atrophy and/or hyperreflective foci in the outer retina on HD-OCT.^[120]^

Cases are often assumed “chronic” in the presence of non-resolving fluid or with degenerative RPE changes without obvious fluid.^[121]^ A recent study provided six retina specialists with anonymous clinical data to correlate with multimodal imaging including OCT, SW-AF, FA, and ICGA for 100 patients. Significant discrepancy between diagnostic descriptions, especially among chronic CSCR cases, was noted, with 36 different terms used by graders.^[121]^ Small amounts of fluid could be easily identified on OCT. However, a “subclinical” term was frequently used when hypo-AF on FAF was present without fluid on OCT, highlighting the importance of FAF for diagnosis when neither FA nor OCT show clinical activity.

A study by Han et al described five patterns of acute CSCR on FAF: blocked FAF, with no changes or uniform hypo-AF in the region of SRF; mottled FAF, with grainy AF signal when compared with normal surrounding background AF; hyper FAF when compared with surrounding AF; hyper/hypo FAF with mixed areas of AF; and descending tract, with inferiorly directed hypo-AF originating in the macula.^[122]^ Duration of symptoms was shortest in the blocked FAF group, followed by mottled FAF, hyper FAF, hyper/hypo FAF, and descending tract. Additionally, blocked FAF had the best mean VA, which is reasonable given likely shorter duration of SRF in this group. The presence of EZ disruption, OS elongation, and RPE proliferation on HD-OCT were more likely with increasing duration of symptoms; all “blocked FAF” eyes had fully intact EZ layers. Increased duration of SRF likely resulted in variable accumulation of shed photoreceptor outer segments and resultant increased FAF.

At the final visit in a large study of chronic CSCR, FAF showed variable morphologies, including mottled hyper-AF and hypo-AF changes, multifocal posterior pole lesions, and inferior gravitational tracts.^[123]^ These gravitational tracts were noted in 49% of eyes, which was similar to the rate reported in another study.^[119]^ Foveal hypo-AF was seen in 75% of eyes, and foveal AF change was the only FAF feature that correlated with poorer VA at last visit.

In a study by Imamura et al, visual acuity decreases were significantly associated with age, confluent hypo-AF in the macula or peripapillary region, and granular hypo-AF in a multivariate regression analysis.^[124]^ Another study evaluated macular FAF compared with MP sensitivities and VA in chronic CSCR. The mean retinal sensitivity in regions of iso-FAF (as compared with an extramacular region of normal signal) was normal, while it was reduced at points with both hyper-AF and hypo-AF.^[125]^

More CSCR abnormalities can be imaged with UWF-FAF than traditional FAF, with 57% of CSCR eyes in one study containing extensive peripheral retinal involvement.^[119]^ Peripheral hyper-AF lesions may indicate activity in the absence of macular involvement and warrant additional imaging.

Iovino et al used the term RPE aperture to describe hypo-AF areas within PEDs in CSCR.^[126]^ Multimodal imaging demonstrated that RPE apertures corresponded to areas of RPE interruption or attenuation mostly at the apex but also at the bases of the PED, and both FA and OCTA showed no evidence of CNV. These apertures should be distinguished from RPE rips, which assume neovascularization, in order to prevent unwarranted treatment.

Although most studies used SW-AF, there is suggestion that NIR-AF may be more sensitive in detecting retinal abnormalities in CSCR.^[118]^ In addition, regions of hypo-AF on NIR-AF correlated with the site of RPE detachment with or without leakage on FA. However, whether or not NIR-AF provides additional information for clinical decision-making is unclear, and most data support a multimodal approach with HD-OCT, FAF, and FA to guide treatment.

### Retinal Dystrophies

Many retinal dystrophies are particularly suited for evaluation with FAF due to the degeneration of photoreceptor and RPE with or without the accumulation of LF. AF patterns have been shown to aid diagnosis in a multitude of dystrophies including STGD/fundus flavimaculatus,^[127]^ Best vitelliform macular dystrophy (BD),^[128]^ retinitis pigmentosa/rod-cone dystrophies (RP),^[129]^ central areolar choroidal dystrophy (CACD),^[130]^ and the pattern dystrophies.^[131]^ Indeed, a deep-learning neural network was able to distinguish between STGD, BD, RP, and healthy retinas with an accuracy of 95% based on FAF alone.^[132]^

However, significant phenotypic overlap can exist among many different genotypes.^[133]^ With improved speed, lower costs, and high accuracy of newer genetic panels, the genotype of the dystrophy has become the paradigm for diagnosis of inherited retinal diseases, especially with the introduction of targeted gene therapies.^[134]^ Regardless, multimodal imaging, especially FAF given its cost-effectiveness and accessibility, has become paramount in monitoring disease progression for prognostication and for clinical trial outcomes.^[135,136]^

#### Stargardt disease (STGD)

The most common juvenile retinal dystrophy, STGD is a phenotype characterized by variable macular atrophy with white–yellow deep retinal lesions in the posterior pole and extending to the mid-peripheral retina.^[137]^ These so-called “pisciform” flecks are variably hyper-AF due to the buildup of RPE LF.^[138]^ It is classically associated with autosomal recessive (AR) mutations in the *ABCA4* gene although autosomal dominant (AD) *ELOVL4 *and *PRPH2/RDS *have been reported.^[139,140]^

Fundus of Stargardt patients display generalized increased AF which reaches a peak value at a certain age and then declines, with the development of dark flecks and/or atrophic changes.^[141]^ The age of this AF ceiling has been shown to depend on both the severity of *ABCA4* mutation as well as gene affected. Sparing of the peripapillary retina on SW-AF is characteristic of STGD. Lois et al showed normal, low, high, or mixed FAF patterns in the macula could be found [127,Figure 6]. Lower macular AF values were associated with more atrophy and peripheral rod and cone dysfunction. The subclinical buildup of LF results in increased qAF levels in regions of normal-appearing retina that may precede visual impairment.^[142]^ One case of presumed STGD demonstrated normal qAF and was thereafter found to have X-linked retinitis pigmentosa.^[143]^

Although SW-AF is the predominant modality in existing clinical trials and cross-sectional studies, both SW-AF and NIR-FAF sufficiently image the abnormalities in STD. Cicinelli et al found that while NIR-FAF and SW-AF agreed on baseline assessments, they measured different rates of progression on follow-up and were therefore not interchangeable.^[144]^

Three patterns were described by Klufas et al^[145]^ on UWF-FAF imaging. Type I consisted of only macular lesions. Type II consisted of macular atrophy with variable peripheral FAF flecks and atrophy. Type 3 contained both macular and peripheral atrophy with three subtypes based on extent of atrophy. Peripheral abnormalities outside of the standard 55° FAF imaging were noted in 72.4% of cases. These included more extensive atrophic or hyper-AF flecks, a posterior polar zone of increased AF in a circular pattern outside of the arcades, and occasional semicircular or zonal abnormalities.

Longer SW-AF lifetimes for individual flecks on FLIO have been noted after one year of follow-up.^[46]^

#### Best disease (BD) and vitelliform maculopathies

Conditions characterized by the accumulation of subfoveal vitelliform material include BD, autosomal recessive bestrophinopathy (ARB), adult onset vitelliform macular dystrophy (AOVMD) (one of the pattern dystrophies), and both idiopathic and paraneoplastic acute exudative polymorphous vitelliform maculopathy (AEPVM). Vitelliform material was shown to be formed from shed photoreceptor debris and LF accumulation in the subretinal space.^[146]^

One of the more common early onset macular dystrophies, BD results from AD mutations in *BEST1*, the gene encoding bestrophin-1, whose product localizes to the basolateral plasma membrane of the RPE.^[147]^ Progression of BD is classically described by five stages with variable rates of progression: previtelliform (subclinical), vitrelliform, pseudohypopyon, vitelliruptive, and atrophic/cicatricial.

Parodi et al described six main FAF patterns in BD: normal, hyper-autofluorescent, hypo-autofluorescent, patchy, spoke-like, and multifocal.^[128]^ The hyper-AF pattern on SW-AF was associated with earlier stages and the best BCVA, while the hypo-AF pattern was seen more often in later stages and the poorest BCVA. Areas of hyper-AF on SW-AF generally correspond to subretinal deposits of vitelliform material on HD-OCT. The pseudohypopyon stage exhibits hyper-AF in the region of the vitelliform material, with hypo-AF elsewhere on the SW-AF. The NIR-AF images in this stage show generalized hypo-AF with more hyper-AF foci. In the vitelliruptive phase, both hyper-AF and hypo-AF zones can be noted on SW-AF and NIR-AF. The atrophic stage is marked by mostly absent AF signal on both SW-AF and NIR-AF.^[148,149]^

Interestingly, preclinical central hypo-AF on NIR-AF in patients with confirmed *BEST1* mutations were noted before any changes on SD-OCT or SW-AF evolved.^[148,150]^ Parodi et al suggest this relates to RPE dysfunction and impaired melanin exocytosis and dispersion, leading to hypo-AF in NIR-AF.^[128,150]^ Areas outside the central lesion do not display increased qAF levels, as opposed to STGD.^[151]^

AF findings in ARB are similar but often more extensive and variable, especially on UWF-FAF imaging.^[152]^ These include areas of vitelliform deposition with hyper-AF, as well as zonal swaths of hyper-AF and/or mottled hypo-AF. The hyper-AF regions may correspond to outer retinal thickening and subretinal depositions, or to outer retinal atrophy.^[152]^

Both idiopathic^[153]^ and paraneoplastic^[154]^ AEPVM have been described, and both present with acute vision loss associated with multiple, diffuse, yellow–white, variably shaped deep chorioretinal lesions, and serous macular detachments. In early stages, the serous fluid is not hyper-AF on SW-AF, but as the yellow–white subretinal material develops, it autofluoresces brightly. These lesions can evolve with gravity dependence and dissolution, forming punctate hyper-AF and hypo-AF patterns with eventual normalization of the FAF pattern.^[153,155]^

#### Pattern dystrophies

The pattern dystrophies are grouped as such due to patterns of abnormal LF accumulation and RPE damage in the posterior pole.^[156]^ They are genetically heterogeneous but are most frequently caused by *PRPH2* gene mutations. The gene product is an integral membrane protein that functions in photoreceptor outer segment morphogenesis.^[157]^

Although classified as a pattern dystrophy, AOVMD is phenotypically diverse and shares many features with the bestrophinopathies. It usually presents in middle age with bilateral, subfoveal, vitelliform lesions less than one-half DD in width but may occasionally be multifocal.^[131]^ Parodi et al examined AOVMD with SW-AF and NIR-AF. On SW-AF, maculas with AOVMD displayed a normal pattern, focal hyper-AF, or patchy AF. They found BCVA to be decreased compared with controls in all AOVMD, with the lowest BCVA in the patchy group. With NIR-AF, the fovea can display central hypo-AF with surrounding variable hyper-AF, occasionally with a central hyper-AF lesion.

Multifocal pattern dystrophy (MPD) simulating fundus flavimaculatus/STGD shows similar findings to *ABCA4*-related STGD, although it usually occurs at an older age. On SW-AF, multiple yellow flecks in the peripheral macula are hyper-AF and may be surrounded by small adjacent hypo-AF zones.^[158]^ Atrophy of the posterior pole is evident usually at ages past 50, much older than STGD in which atrophy usually presents by adolescence.

Butterfly pattern dystrophy is characterized by a butterfly wing-shaped pattern of pigment deposition at the macula. A mixed pattern of SW-AF can be noted, with focal hyper-AF corresponding to the yellowish LF deposits or pigmented RPE hypertrophy, with hypo-AF in surrounding atrophic areas.^[156,159]^

Reticular pattern dystrophy is rare and presents with net-like pigmentation of the macula. This is delineated on SW-AF with hyper-AF with surrounding hypo-AF.^[160]^

Fundus pulverulentus is very rare pattern dystrophy noted by coarse pigment mottling in the macula. Multiple hypo-AF spots with interposed reticular hyper-AF lesions may be present.^[161]^

#### Central areolar choroidal dystrophy (CACD) 

CACD is an inherited retinal dystrophy causing dysfunction of photoreceptors and visual acuity usually between ages 30 and 60, resulting in a well-demarcated macular area of outer retinal, RPE, and choriocapillaris atrophy.^[130]^ It is usually AD,^[162]^ with *PRPH2 *mutations being the most common, although AR cases have been reported.^[163]^

Differentiation between CACD and GA from AMD can be difficult and is often disregarded given the extreme difference in prevalence. Four clinical stages of increasing chorioretinal damage are present in CACD.^[164]^ Stages 1 and 2 may mimic intermediate AMD. A speckled pattern of hypo- and hyper-AF on SW-AF may present that is more well-demarcated and more regular than in eyes with AMD, with less extramacular extension of lesions. Stages 3 and 4 are marked by central GA. This GA in CACD is more sharply demarcated and round to oval as opposed to patchy and more ill-defined GA in AMD. Additionally, FAF changes in AMD often extend beyond the macula and are less well-demarcated than in CACD, and HD-OCT will demonstrate sub-RPE deposits surrounding the GA.

#### Retinitis pigmentosa (RP)/Rod–cone dystrophies

RP is a blanket term given to hereditary retinal dystrophies that feature rod and cone photoreceptor degeneration. It affects approximately 1 in 4,000 individuals worldwide and may feature AD, AR, or X-linked inheritance.^[165]^ Full-field ERG is the gold standard diagnostic test, but in more advanced disease this test loses clinical utility. The widespread and often unique outer retinal and RPE manifestations lend themselves well to FAF imaging.

A characteristic hyper-AF parafoveal ring may be seen in many genotypes of RP on both SW-AF and NIR-AF [129, 166–169, Figure 7]. It is thought to represent an area of increased photoreceptor phagocytosis and LF accumulation. This ring may often constrict over time as the disease progresses, as opposed to cone–rod dystrophies in which the ring may expand.^[170]^ This constriction is marked by a decrease in EZ band diameter on HD-OCT and suggests progression of structural disorganization of photoreceptor.^[171]^ Moreover, retinal sensitivity on MP and both static and dynamic perimetry has been shown to be relatively preserved within the AF ring and impaired outside the ring.^[172]^ Retinal sensitivity correlated well with outer retinal thickness on HD-OCT but not with total or inner retinal thickness.

Murakami et al categorized three SW-AF ring patterns with RP: abnormal ring AF (most common), abnormal central AF, and neither abnormal ring nor central AF.^[166]^ Robson et al demonstrated that radius of the hyper-AF ring was linearly correlated with pERG amplitude,^[173]^ that is, a larger ring showed greater response on pERG, presumably because the hyper-AF ring demarcates the area between markedly abnormal peripheral retina and more normal central retina. Dowd-Schoeman et al evaluated 31 patients with genotype-confirmed RP with various FAF modalities and described macular patterns of central foveal hyper-AF, perifoveal ring hyper-AF, macular ring hyper-AF, and a bull's eye AF. They further noted extramacular AF patterns of a mid-peripheral hyper-AF ring, extramacular hyper-AF spots, patchy hypo-AF, and diffuse peripheral hypo-AF.^[174]^ Oishi et al imaged 75 patients with various RP genotypes using UWF-FAF.^[175]^ They described three types of macular FAF: type 2 demonstrated ring-shaped perifoveal hyper-AF, type 3 showed abnormal foveal hyper-AF, and type 1 showed neither finding. Regardless of peripheral pathology, type 3 eyes had worse VA than type 1 or 2. The percentage of abnormal peripheral hypo-AF correlated well with visual field area, VA, duration of symptoms, and mean deviation (MD) on static perimetry.

Schuerch et al demonstrated with non-UWF imaging that SW-AF qAF values internal to the ring were abnormal in only 29% of patients, while qAF values external to the ring were abnormal in just 53% of eyes.^[170]^ Another study by Duncker et al noted much lower NIR-AF signal outside of the ring compared to inside the ring, and the inner border of the ring was closer to the fovea on NIR-AF than SW-AF.^[176]^ The inner NIR-AF border corresponded to a location where the EZ band was partially intact on HD-OCT, suggesting NIR-AF can detect RP progression prior to the development of SW-AF-sensitive fluorophores.

Dysli et al evaluated FLIO patterns in patients with RP.^[177]^ Longer lifetimes were found in areas of photoreceptor atrophy and even longer lifetimes in areas of photoreceptor and RPE atrophy. The perifoveal ring of prolonged lifetimes was wider than that seen on qualitative SW-AF. However, although the measured FLIO values just outside the ring were statistically longer than those just inside of the ring, they were not significantly longer than healthy eyes. This is in line with the above findings of abnormal qAF findings external and internal to the ring in only a subset of patients.^[170]^

An autofluorescent ring is not specific to RP, however, and can be found in pigmented paravenous retinochoroidal atrophy (PPRCA),^[178]^ macular dystrophies, Leber's congenital amaurosis (LCA),^[179]^ Best disease, some cone–rod dystrophies, and X-linked retinoschisis (RS).^[180]^ This shared findings suggests common mechanisms of retinal degeneration in certain retinal dystrophies.

As a pan-retinal degeneration, RP may be better imaged with UWF modalities. Peripheral hypo-AF may be punctate/mottled or nummular/patchy, with more extensive patchy changes seen in older patients or those with longer symptom durations.^[175]^ The cumulative area of hypo-AF on UWF-FAF were significantly correlated with Goldmann perimetry scotomata in another study,^[181]^ but areas of peripheral mottled FAF did not necessarily correlate with VF defects. Retinal sensitivity is better correlated to outer retinal thickness and therefore photoreceptor damage,^[182]^ which is often demarcated by the hyper-AF macula ring, and severe peripheral VF defects can be seen in the absence of RPE loss and marked hypo-AF.^[183]^ Other studies found female carriers of X-linked RP with *RPGR *mutations to demonstrate variable radial FAF patterns that can extend to the periphery.^[184,185]^ An inferonasal boundary of abnormal FAF patterns may be noted in patients with RP or STGD and is thought to be related to closure of the optic fissure.^[186]^

Bietti's crystalline dystrophy (BCD) is an RP phenotype caused by AR mutations in *CYP4V2.* ^[187]^ Along with diffuse retinal and RPE degeneration, it is marked by crystalline deposits in the retina and occasionally the cornea. These deposits are not easily detectable on SW-AF, and they may disappear with progressive chorioretinal degeneration and while they are hallmarks of BCD, they can be seen in other retinal conditions. Crystalline deposition on fundus photographs slightly precedes mottled hypo-AF RPE degeneration on SW-AF that can begin in the posterior pole and peripapillary region.^[188]^ This eventually leads to diffuse hypo-AF with remaining isoAF irregular islands of RPE. However, hypo-AF and hyper-AF lesions are not spatially correlated with the crystalline deposits on color imaging,^[189,190]^ and NIR-R appears to visualize the crystalline deposits best, although this is also variable.^[191]^ Sclerotic choroidal vessels are typical in BCD and underlie regions of chorioretinal atrophy that can be seen on color imaging. These sclerotic vessels have been shown to be hyper-AF on SW-AF, while non-sclerotic choroidal vessels remain hypo-AF.^[190]^

Mutations in the *CRB1* gene can lead to both LCA and RP with distinctive FAF features. Typical findings of peripheral bone-spicule retinal and RPE degeneration with or without hyper-AF macular rings can be found. However, hyper-AF optic disc drusen and a peculiar peri-arteriolar sparing of the RPE with normal AF can be seen.^[192]^ Peripapillary and peripheral hamartomas have been reported with variable SW-AF patterns.^[192,193]^

PPRCA is a degenerative phenotype marked by retinal, RPE, and choriocapillaris atrophy with pigmentation primarily along the retinal veins. Inflammatory, infectious, genetic, and idiopathic etiologies have been described, but the unifying pathogenesis remains unclear.^[194]^ Paravenous hypo-AF from RPE atrophy and pigment clumping can be found, with adjacent zones of hyper-AF [195, Figure 8]. These zones of hyper-AF may be found along peripheral veins and may precede frank RPE atrophy and pigment clumping.^[196]^

Certain FAF patterns have been thought to be pathognomonic for certain RP genotypes. For example, a double concentric hyper-AF ring was first described in *NR2E3*-linked RP^[197]^ and attributed to this mutation, but was thereafter also noted in *USH2A*-linked RP.^[198]^ In a study by Trichonas et al, the pattern of peripheral UWF-FAF was noted to differ among patients with certain genetically confirmed RP mutations.^[199]^ Distinct changes such as the double hyper-AF ring with *USH2A* or diffuse peripheral hypo-AF and dark appearance with *USH2A *or *PRPH2/RDS *mutations were demonstrated. However, a recent study also noted the double hyper-AF ring in a patient with *RHO*-linked RP and a patient with *RPGR*-linked RP, as well as significant overlap in other FAF patterns among genotypes.^[174]^ Although many patterns of FAF have been described in RP, and some show predilection for certain genes, it is difficult to determine the RP genotype by FAF pattern alone. However, FAF can be helpful in differentiating RP from other conditions in patients with unexplained visual acuity or visual field loss with or without abnormal findings on fundus exam. FAF findings in RP are generally symmetrical and often include hypo-AF spots or patches in the midperiphery with or without macular AF changes. In addition, FAF is helpful in assessing progression of the disease over time; this may include an increase in hypo-AF spots and a reduction in the size of macular hyper-AF ring.

### Retinal Toxicities

#### Hydroxychloroquine (HCQ)

HCQ (brand name Plaquenil) is commonly used for treatment of systemic lupus erythematosus, rheumatoid arthritis, and other inflammatory systemic and dermatologic conditions. Patients using ≤ 5 mg/kg real weight daily have < 1% risk in the first 5 years of therapy and < 2% at 10 years.^[200]^ After baseline evaluation, routine annual screening after five years is recommend by the American Academy of Ophthalmology (AAO), with SW-AF recommended as one of the ancillary screening tests.^[201]^ In non-Asians, HCQ toxicity manifests with parafoveal hyper-AF rings, corresponding to photoreceptor degeneration, that eventually becomes less hyper-AF or even hypo-AF with progression to RPE atrophy.^[202]^ In Asians, there is a higher chance for additional pericentral, peripheral macular damage, or even pericentral changes alone.^[203]^ Patients with pericentral damage also tend to have longer duration of HCQ therapy and larger cumulative doses.

The maculopathy may be asymmetric, often starting inferiorly and progressing circumferentially, but may be more symmetric on NIR-FAF. Early changes may be more detectable on qAF as well.^[204]^ Prolonged FLIO lifetimes have been noted in a parafoveal distribution corresponding to regions of toxicity.^[205]^

Despite this, HD-OCT has been reported as more sensitive for detecting HCQ-related structural damage than either SW-AF or NIR-AF,^[206]^ and current guidelines still recommend HD-OCT and VF as primary screening modalities.

#### Pentosan maculopathy

A pigmentary maculopathy associated with pentosan polysulfate sodium (PPS), a commonly used treatment of bladder pain and interstitial cystitis, was recently described by Pearce et al.^[207]^ Findings included subtle vitelliform deposits and patchy paracentral RPE atrophy that on SW-AF were far more apparent as mottled, irregular patterns of FAF in the posterior pole. Further studies demonstrated a wider phenotypic spectrum, from subtle pattern-like macular changes to confluent pattern of hyper-AF and hypo-AF spots and reticular changes, to widespread nummular chorioretinal atrophy.^[208,209]^ The hyper-AF lesions localized with yellow deposits and hyperpigmented spots on color imaging. These hyperpigmented spots localized to focal thickening of the RPE on HD-OCT. Most patients show relatively preserved VA in the range of 20/20 to 20/60 but may have subjective visual complaints such as blurring or nyctalopia.

While toxic dose thresholds are still unclear, Wang et al noted no affected patient with cumulative doses < 1500 gm, and in prior studies all affected patients except one reported at least a 500 gm cumulative dose.^[207,208]^ A mathematical analysis of one cohort found the greatest correlation with standardized cumulative PPS dose adjusted to weight,^[210]^ with 75% (*n* = 3) of patients with accumulated dose of > 20 gm/kg demonstrating obvious hypo-AF defects on SW-AF. Prevalence of PPS-toxicity is so far reported as approximately 20%.^[209,211]^

#### Deferoxamine mesylate (DFO)

DFO is an iron-chelating compound given for treatment of chronic iron overload in conditions such as β-thalassemia that require frequent blood transfusions. Retinal complications of long-term use include macular and peripheral pigmentary changes accompanied by mildly reduced VA and diminished ERG amplitudes.^[212]^ Patterns of SW-AF abnormalities are far more prominent than color imaging and have been categorized into minimal changes, focal hyper-AF with a background of reticular hypo-AF, patchy hyper-AF in a pattern-like distribution, or a diffuse speckled pattern of FAF changes.^[212]^ Studies noted more severe visual abnormalities with the patchy or speckled patterns.^[213,214]^ Pattern-like changes described include a butterfly-like, fundus flavimaculatus-like, fundus pulverulentus-like, and vitelliform-like distributions.

### Miscellaneous Conditions

#### Pseudoxanthoma elasticum (PXE)

PXE is a recessive genetic disorder, marked by mutations in *ABCC6*,^[215]^ that affects the skin and connective tissues, including ocular structures. Ophthalmic findings include peau d'orange, optic nerve head drusen, RPE atrophy, angioid streaks, “comet-tail” deposits, and CNV.^[216]^ Macular SW-AF changes include hypo-AF subretinal deposits similar to SDD on HD-OCT, hypo-AF subretinal deposits that appear yellow–brown and cause a pattern dystrophy-like appearance, and hyper-AF subretinal deposits that may be accompanied by SRF. While dark orange–red on color imaging, angioid streaks appear hypo-AF with isoAF specks and are often accompanied by hypo-AF peripapillary atrophy. Progressive RPE atrophy with or without CNV formation is hypo-AF, often with intervening hyper-AF specks, and may extend from the macula and peripapillary area into the periphery. Calcification of Bruch's membrane posteriorly, termed “coquille d'oeuf” (eggshell), is peripherally bordered by the classic peau d'orange. This calcification may be very subtly hypo-AF and more detectable by decreased qAF.^[217]^ Comet lesions and optic disc drusen are hyper-AF.^[218]^ Widespread posterior pole atrophy may obscure many classic findings, and UWF-FAF may allow detection of peripheral lesions to aid diagnosis.^[219]^

#### Macular telangectasia type 2 (MacTel)

MacTel is an idiopathic bilateral condition causing characteristic macular changes and affecting the juxtafoveolar capillary network.^[220]^ Retinal atrophy, cavitation, crystal deposition, microaneurysms and vascular abnormalities with leakage, and CNV may result with disease progression. Impaired storage of pigments such as lutein and zeaxanthin, purportedly by Muller cells, results in depletion of macular pigments and increased central macular AF^[221]^ as well as increased FLIO.^[222]^ This pigment loss starts temporally and spreads throughout the fovea, but pigment clumping may cause discrete plaques that are hypo-AF. The presence of increased macular AF or clumps of pigment causing decreased AF were found to be associated with worse VA at baseline and were predictive of slow visual decline at two years, although the natural history of the disease is slowly progressive.^[223]^ Retinal crystals, extent of involvement, and angled dipping vessels (right-angle venules) are better represented on color imaging than FAF.^[224]^ However, SW-AF can detect subtle, usually temporal hyper-AF that can precede HD-OCT, MP, and angiographic changes,^[225]^ but whether this is clinically useful given current lack of effective treatments is to be seen.

#### Rhegmatogenous retinal detachment (RRD) and retinoschisis (RS)

While HD-OCT imaging can clearly delineate RRD versus RS, technical challenges of capturing peripheral images limit its clinical utility. The advent of UWF imaging provided quick and reliable monitoring of peripheral pathologies but without the depth resolution of OCT. A number of studies have examined the FAF signal in RRD, RS, and RS detachment (RSD) given previous work noting FAF changes related to retinal layer separations and metabolic changes.^[57]^

Witmer et al demonstrated hypo-AF over the detachment in all eyes, with a hyper-AF leading edge or posterior margin (PM) in all macula-involving RRDs and 75% of macula-on RRDs.^[226]^ This hyper-AF PM corresponded to shallow SRF leading up to the frank RRD. After PPV, disappearance of the hyper-AF PM with demonstration of a demarcation line often occurred. Another study examined FAF changes after primary SB and noted FAF changes in the area of cryopexy depending on the amount of RPE destruction, and buckling elements produced hypo-AF shadows at their posterior edge, with radial hyper-AF streaks approximately 50% of the time.^[227]^

Navaratnam et al expanded on these findings and included eyes with RS and RSD. Although finding similar changes with acute RRD, they noted isoAF or hypo-AF in areas of chronic RRD ( > 2 weeks), with variable FAF patterns at their PM.^[228]^ All eyes with RS showed no or minimal AF change over the main RS cavity, while the PM was variable. Pooled analysis found that hypo-AF in RRD had a positive predictive value (PPV) of 95% and negative predictive value (NPV) of 76%, while hyper-AF in RSD indicating SRF had a PPV of 100%.

Francone et al correlated HD-OCT with UWF-FAF in patients with RS complicated by RRD and also found mostly isoAF change over the RS cavity with a hypo-AF posterior border in bullous RS. However, hyper-AF at or extending past the PM of the RS corresponded to SRF or full-thickness RRD on HD-OCT in all eyes.^[229]^

Hypo-AF over the RRD in these studies was purported to result from blockage of RPE signal from edematous retina and SRF, while the hyper-AF PM was suggested to result from buildup of fluorophores in either photoreceptor outer segments or RPE from impaired metabolism. Careful examination of RS with serial UWF-FAF imaging or peri-operative UWF-FAF imaging of RRDs can be useful to detect progression of SRF or conversion of schisis cavities into full-thickness detachments.

##  SUMMARY

FAF allows noninvasive and *in vivo *topographical imaging of retinal metabolic changes and general health of the photoreceptor and RPE layers. Advances in imaging technologies have demonstrated more peripheral FAF changes as well as earlier detection of macular metabolic changes that can be invaluable for clinical decision-making. Several imaging platforms are available, each with their advantages and disadvantages, and careful consideration should be made both in system utilization and imaging interpretation based on published findings with each modality. While SW-AF has historically been most studied, further improvements and accessibility for other techniques such as NIR-FAF, qAF, and FLIO may increase diagnostic and monitoring capabilities.

##  Financial Support and Sponsorship 

This review has been supported, in part, by an unrestricted grant to the Department of Ophthalmology of the University of Southern California from Research to Prevent Blindness, New York, NY.

##  Conflicts of Interest

There is no conflict of interest.
